# The Use of Janus Kinase Inhibitors in Axial Spondyloarthritis: Current Insights

**DOI:** 10.3390/ph15030270

**Published:** 2022-02-22

**Authors:** Eric Toussirot

**Affiliations:** 1 INSERM CIC-1431, Centre d’Investigation Clinique, Pôle Recherche, CHU de Besançon, 25000 Besançon, France; etoussirot@chu-besancon.fr; 2 Rhumatologie, Pôle PACTE (Pathologies Aiguës Chroniques Transplantation Éducation), CHU de Besançon, 25000 Besançon, France; 3 Département Universitaire de Thérapeutique, Université de Bourgogne Franche-Comté, 25000 Besançon, France; 4 INSERM UMR1098 Right “Relations Hôte Greffon Tumeurs, Ingénierie Cellulaire et Génique”, Université de Bourgogne Franche-Comté, 25000 Besançon, France

**Keywords:** axial spondyloarthritis, JAK/STAT, JAK inhibitors, Janus kinase

## Abstract

Current pharmacological treatments of axial spondyloarthritis (axSpA) are limited to non-steroidal anti-inflammatory drugs (NSAIDs) and biological agents, including TNFα inhibitors and IL-17 inhibitors. Despite the availability of these agents, many patients either fail to respond adequately, lose their initial therapeutic response over time, or develop undesirable side effects, thus highlighting the need for new treatment options. Janus kinase (JAK) and signal transducers and activators of transcription (STAT) are a group of intracellular kinases that play a role in the signaling pathway induced by cytokines and certain growth factors associated with the inflammatory process of axSpA. There are several lines of evidence implicating the JAK–STAT pathway in the pathophysiological process of axSpA, including genetic data, the use of certain JAK in the intracellular signal of specific cytokines involved in axSpA (IL-23, IL-22, and IL-6), and data from experimental models of SpA. This provides a rationale for the assessment of JAK inhibitors (JAKi) in clinical trials with patients with axSpA. In this review, we examine the role of JAK–STAT signaling in the pathogenesis of axSpA and summarize the results from recent clinical trials of JAKi (tofacitinib, upadacitinib, and filgotinib) in patients with axSpA.

## 1. Introduction

Spondyloarthritis (SpA) encompasses a group of chronic inflammatory diseases presenting with common clinical, genetic, imaging, and pathophysiological features. Clinical manifestations of SpA include spinal inflammation and peripheral arthritis, as well as enthesitis and dactylitis. Spondyloarthritis is subdivided into axial and peripheral forms, according to the predominant clinical manifestations. Axial spondyloarthritis (axSpA) primarily affects the axial skeleton, i.e., the sacroiliac joints (SIJ) and the spine [1]. Radiographic changes of the SIJ are a hallmark of the disease and enable the diagnosis of ankylosing spondylitis (AS) according to the modified New York criteria [2]. The Assessment of SpondyloArthritis international Society (ASAS) has developed a set of criteria for the recognition of patients with early axSpA that includes evidence of sacroiliitis visible by magnetic resonance imaging (MRI), chronic back pain, HLA-B27 positivity, and other non-articular symptoms [3]. These criteria distinguish the radiographic (r-axSpA, formerly AS) and non-radiographic (nr-axSpA) forms of axSpA, according to the presence or absence of structural changes of the SIJ on pelvic X-rays. Spondyloarthritis is also characterized by well-known extra-articular manifestations that may affect the skin (psoriasis), the eye (acute anterior uveitis), and the gut (inflammatory bowel diseases) [4].

Both the articular and extra-articular manifestations are key features for the diagnosis and the management of SpA. Indeed, the therapeutic management of SpA is challenging, considering the heterogeneity of the disease. Consequently, treatment selection is influenced by the dominant clinical features [5]. For axSpA, treatment options include NSAIDs as the first line, and biological agents in the case of failure or intolerance to NSAIDs [6]. Currently, two classes of biologics are available and licensed for the treatment of both r-axSpA and nr-axSpA, namely TNFα inhibitors (TNFi) and IL-17 inhibitors (IL-17i). These biological agents, especially TNFi, have dramatically changed the therapeutic landscape of axSpA by demonstrating major improvements in key disease domains and by improving the quality of life for patients [7]. Despite this high level of efficacy, treatment options remain limited in axSpA, and a significant proportion of patients do not have adequate response (i.e., inactive disease or low disease activity), or they may lose their initial response over time. In addition, some patients do not tolerate these treatments and can experience undesirable side effects, including infections. For these reasons, alternative drugs with another mechanism of action are required in axSpA [5].

Janus kinase (JAK) and signal transducer and activator of transcription (STAT) are key molecules involved in the signaling of pro- and anti-inflammatory cytokines [8]. Different cytokines are involved in the pathogenesis of axSpA, including TNFα and IL-17 [9]. The IL-23/IL17 pathway, a major player in SpA immune mechanisms, is partly controlled by JAK [10]. Thus, JAK inhibitors (JAKis) may represent a new drug class for the management of axSpA. In this review, we analyze the rationale for using JAKi and summarize results of clinical trials of these small synthetic drugs in axSpA.

## 2. Key Mechanisms of Disease Pathogenesis in axSpA

Numerous interrelated etiological factors are involved in the pathogenesis of SpA. It is thought that both genetic and environmental factors combine to elicit a chronic inflammatory response [4]. Both the innate and adaptive immune system contribute to this chronic inflammation. Triggering factors that may initiate inflammation in axSpA include infectious agents, alterations of the gut microbiome, and mechanical stress [11]. The primary site of inflammation of axSpA is the entheseal structures [12], the site where the tendons and ligaments are attached to the bone surface, with secondary involvement of adjacent structures, corresponding to the so-called synovio–entheseal complex [13]. The sacroiliac joints are another site of initial inflammation in axSpA. An infiltrate composed of macrophages and activated T cells leads to the production of inflammatory cytokines including IL-1, IL-10, TNFα, and IL-17, contributing to recruitment and activation of immune cells, ultimately resulting in joint/enthese damage [14,15]. SpA is further characterized by osteoproliferative changes, with bone formation at the site of inflammation, a phenomenon that follows the inflammatory phase, leading to the well-described enthesophytes, bone formation, and fusion of the SIJ [16]. TNFα has been identified as a key driver of inflammation in axSpA, prompting the clinical development of specific effective agents that neutralize its biological effects [17]. The IL-23/Th17 pathway was next identified as another major determinant in SpA pathogenesis [18]. IL-23, together with IL-6, contributes to the differentiation of Th17 lymphocytes, which produce IL-17, as well as TNFα and IL-22. All of these cytokines play a role in local inflammation and destructive changes (erosions), as well as in new-bone formation [19].

## 3. The JAK/STAT Pathway

**Figure 1:**
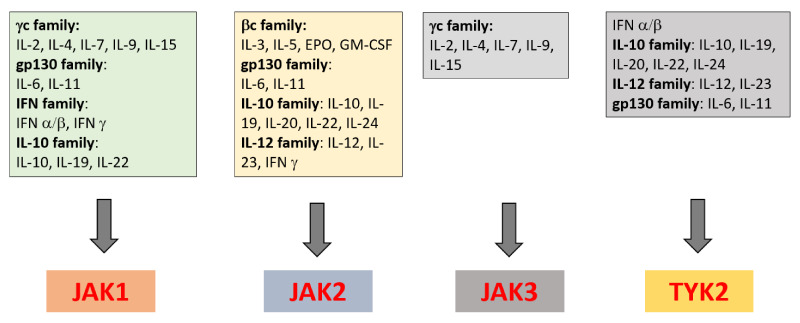
Use of the members of the Janus kinases (JAKs) (one or more JAK) by different cytokines.

JAK is a family of molecules with four members: JAK1, JAK2, JAK3, and TYK2. These tyrosine kinases are able to phosphorylate tyrosine residue on themselves or on adjacent molecules, such as STAT. STAT is a family of transcription factors that includes seven members: STAT1, STAT2, STAT3, STAT4, STAT5a, STAT5b, and STAT6. The JAK/STAT pathway is involved in the signaling of various molecules, including cytokines, interferons (IFN), growth factors, and hormones (Figure 1) [8,20]. All of them exert their functions through type I and type II receptors, which do not possess an enzymatic kinase domain. Instead, each type I and type II cytokine receptor is associated with a pair of JAK that is required for signaling. The binding of a cytokine to its receptor leads to autophosphorylation of JAK, which, in turn, phosphorylates sites on the intracellular domain of the cytokine receptor, leading to the formation of a binding domain to STAT. STAT is then phosphorylated, and subsequently migrates to the nucleus, where it regulates gene expression [21].

## 4. The Implication of the JAK/STAT Pathway in Spondyloarthritis

Different cytokines signal by using different pairs of JAKs. For instance, the γ-common cytokines (including IL-2, IL-4, IL-7, IL-15, and IL-21) signal by using JAK1/JAK2, inducing adaptive immune cell differentiation. IFNγ and IL-12 signal through JAK1/JAK2 and JAK2/TYK2, influencing Th1 response and production of TNFα (Figure 1) [20]. IL-23 signals via a JAK2/TYK2 combination, while IL-6 uses JAK1/JAK2 [10,22]. The IL-22 signal is mediated by JAK1/TYK2. IL-7 signaling uses JAK1/JAK3 and induces IL-17 production by mucosal associated invariant T (MAIT) cells [10]. Of interest, TNFα, a key cytokine driving inflammation in axSpA, does not use the JAK/STAT pathway. However, inhibition of JAK2/TYK2 or JAK1/JAK2 modulates its production by the inhibition of IL-12 and IFNγ production [23,24,25]. Genetic studies have identified polymorphisms in JAK2 and STAT3 as susceptibility factors for AS [26]. Polymorphisms in TYK2 can influence susceptibility to different autoimmune/inflammatory diseases, including AS [27]. Furthermore, JAK2 polymorphisms have been described in immune-mediated diseases that are associated with axSpA, namely Crohn’s disease or psoriasis [28]. Collectively, polymporphisms in JAK/STAT argue for a role of this kinase system in the pathogenesis of axSpA. Finally, experimental models of SpA provide additional mechanisms implicating the JAK/SAT pathway in axSpA. Indeed, blockade of JAK/STAT in animal models results in clinical changes in SpA: in an SKG mouse model of SpA induced by curdlan, blockade of JAK/STAT suppressed inflammation and periosteal/entheseal bone formation [29]. JAKi may reduce Th17 response in CD4+ T cells from patients with AS [30]. In the SKG model of SpA, a TYK2 inhibitor was capable of blocking disease progression [31]. In addition, this JAKi may abrogate IL-22 production and partially inhibited IL-17 production by γδ T cells. In the A20 (TNFα induced protein-3)-deficient mouse model, which involves the synovio–entheseal complex, tofacitinib, a pan-JAK inhibitor, significantly reduced enthesitis. Importantly, inflammation in this model is independent of TNFα [32]. Collectively, these experimental data strongly support the involvement of the JAK/STAT pathway in axSpA and also indicate that the best targets among the different JAKs are certainly TYK2, JAK2, and JAK1 [10,23].

## 5. Clinical Trials of JAK Inhibitors in axSpA

Numerous JAKis with various selectivity are in development in inflammatory diseases [21,22]. Biologic disease-modifying anti-rheumatic drugs (bDMARDs) have been shown to be highly effective in axSpA, but these agents target individual cytokines (TNFα or Il-17A). Conversely, the inhibition of JAK is associated with the blockade of a broad range of cytokines, depending on their selectivity. To date, four JAKis (tofacitinib, baricitinib, upadacitinib, and filgotinib) are licensed and approved in the treatment of rheumatoid arthritis (RA). For axSpA, three JAKi (tofacitinib, upadacitinib, and filgotinib) with different selectivity, have been evaluated, and, so far, only tofacitinib and upadacitinib have been licensed for the treatment of AS.

### 5.1. Tofacitinib in axSpA

Tofacitinib inhibits JAK1 and JAK3, and, to a lesser extent, JAK2 [21,33]. In a first phase-two placebo-controlled dose-ranging trial, tofacitinib yielded favorable results in patients with axSpA [34]. In this 12-week study, 207 patients with AS were included and received tofacitinib at 2, 5, or 10 mg twice a day, or they received a placebo. The primary endpoint was ASAS20 (Assessment in Ankylosing Spondylitis 20% improvement) response rate at week 12. Patients on 5 mg twice daily achieved ASAS20 at a higher rate compared to the control group (80.8% vs. 41.2%; *p* < 0.001). However, the primary endpoint was not reached for the two other tofacitinib doses. The secondary endpoints (ASAS40, Bath Ankylosing Spondylitis Disease activity index (BASDAI) 50 (50% improvement in baseline BASDAI), and change in Ankylosing Spondylitis Disease activity Score (ASDAS)) showed greater improvements with tofacitinib 5 and 10 mg twice daily than with the placebo. Of interest, patients with objective signs of inflammation (elevated CRP or bone marrow edema on MRI of the SIJ) experienced greater efficacy and a larger treatment effect with all doses of tofacitinib versus the placebo. Changes in MRI scores (SPondyloarthritis Research Consortium of Canada, SPARCC) were also analyzed: at week 12, the reduction from baseline in SIJ SPARCC scores was significantly greater with tofacitinib 5 and 10 mg compared to the placebo, while the reduction from baseline in spinal SPARCC scores was significantly greater with all tofacitinib doses compared to the placebo. Adverse events were similar across treatment groups [34]. This trial was next completed by a phase-three study enrolling patients with AS and an inadequate response to at least two NSAIDs [35]. Patients were randomized to receive tofacitinib 5 mg twice daily, or placebo, for 16 weeks, followed by an open-label tofacitinib period until week 48. The primary endpoint was the proportion of ASAS20 at week 16. In total, 269 patients were randomized in this trial. At week 16, there was a higher rate of ASAS20 response in the tofacitinib group compared to the placebo (56.4% vs. 29.4%; *p* < 0.0001). The ASAS40 response (a key secondary endpoint) was also greater with tofacitinib versus the placebo (40.6% vs. 12.5%; *p* < 0.0001). Efficacy (according to ASAS20 and ASAS40) was maintained up to week 48 (Table 1) [35]. Finally, a post hoc analysis of patients from the phase-two study described the MRI changes of the SIJ and the spine. This analysis reported minimally important changes (MIC) for SPARCC MRI scores at the SIJ and the spine. A greater proportion of patients achieved MIC with tofacitinib (2, 5, and 10 mg twice daily) versus placebo for SIJ and spine scores (MIC for SIJ = 11.8%, 28.6%, 38.6%, and 29.6% for the placebo and tofacitinib 2, 5, and 10 mg, respectively; MIC for spine = 11.8%, 29.3%, 36.4%, and 40.9% for placebo and tofacitinib 2, 5, and 10 mg, respectively) [36].

### 5.2. Upadacitinib in axSpA

Upadacitinib is a JAK1 selective inhibitor. It was designed to have greater selectivity over JAK2, JAK3, and TYK2 [37]. Upadacitinib was evaluated in axSpA in the SELECT-Axis 1 trial [38]. This was a combined phase-2/3 randomized double-blind placebo-controlled study with two periods. The first part corresponded to the double-blind period over 14 weeks, followed by an open-label extension (period two). In this trial, patients with AS were bDMARD-naive and did not respond adequately to at least two NSAIDs. They were randomized to receive upadacitinib 15 mg daily or a placebo for 14 weeks (period 1), and then patients who completed the first period were eligible for period two, and received open-label upadacitinib up to week 104. The primary endpoint for the first part of the trial was ASAS40 response at week 14. In total, 187 patients participated in the study. The proportion of ASAS40 responders was higher in the upadacitinib group compared to the placebo (52% vs. 26%; *p* = 0.0003). Several secondary endpoints were also significantly improved in the upadacitinib group compared to the placebo, including changes from baseline to week 14 in ASDAS, SPARCC MRI spine score, and the proportion of patients with BASDAI50 and ASAS partial remission. The most common adverse event was elevated creatine phosphokinase (CPK) in 9% of patients under upadacitinib (compared to 2% in the placebo group) (Table 1) [38]. The interim analysis of the SELECT-Axis 1 extension study was published, reporting efficacy and safety data through 1 year [39]. The results showed sustained efficacy over 1 year, and even an increase in ASAS40 response throughout the study: the percentage of ASAS40 responders was higher at week 64 compared to week 14 (72% with non-responder imputation analysis vs. 52%, respectively). Importantly, patients who switched from placebo to the upadacitinib showed a level of response similar to those initially randomized to the upadacitinib group, with a rapid response. At one year, there were no serious infections, major cardiovascular events (MACE), or venous thromboembolic events [39].

### 5.3. Filgotinib in axSpA

Filgotinib is a selective inhibitor of JAK1 [40]. It was evaluated in a phase-two double-blind placebo-controlled trial in axSpA (the TORTUGA trial) [41]. In that study, patients had active AS with inadequate response to at least two NSAIDs. Previous administration of TNFi was allowed. The primary endpoint was the change in ASDAS score from baseline. A total of 116 patients were randomized to receive filgotinib 200 mg daily or a placebo for a 12-week period. The primary endpoint was met, with greater improvement in ASDAS at week 12 in the filgotinib group compared to the placebo group (mean change ± SD: −1.47 ± 1.04 vs. −0.57 ± 0.82; *p* < 0001). Secondary endpoints (including ASAS20, ASAS40, ASAS5/6 (at least 20% improvement in at least five of the following six domains: function, pain, inflammation, patient global, CRP, and spinal mobility), ASAS partial remission, and Bath Ankylosing Spondylitis Functional index (BASFI) at week 12) were also significantly improved in the filgotinib arm as compared to placebo. There was also a significant reduction in spinal and SIJ MRI inflammation scores (using SPARCC). The safety was satisfactory (Table 1) [41]. A post hoc analysis of spine and SIJ MRI was then performed, providing additional information on the changes in inflammatory and erosive lesions. A first analysis described the changes in spinal inflammation of the spine, using the Canada–Denmark (CANDEN) MRI scoring system. This system yields an evaluation of inflammation, fat, erosion, and new-bone formation. The analysis showed a greater reduction with filgotinib versus placebo in total spinal inflammation score, including vertebral bodies, facet joints, and postero-lateral elements. Conversely, there was no significant difference in the change in vertebral body inflammation, spine fat lesion, bone erosion, or new-bone formation between filgotinib and the placebo [42]. A second analysis examined the changes in SIJ structural lesions (erosions, backfill (erosion cavity), fat metaplasia, and ankylosis), using the SPARCC SIJ structural score. The results showed a significant reduction in SIJ erosion score and an increase in backfill score with filgotinib compared to the placebo. This suggests that filgotinib might induce tissue repair, a phenomenon that can be observed as early as 12 weeks [43].

## 6. Discussion

Three JAKis have been evaluated in axSpA, including only patients with a radiographic form. All patients had an inadequate response to at least two NSAIDs, and most were bDMARD-naïve. JAKis were associated with clinical improvement according to commonly used and validated response criteria in axSpA (ASAS20 for tofacitinib, ASAS40 for upadacitinib, and change in ASDAS for filgotinib). JAKis were also associated with improvements in other domains of axSpA, such as function, mobility, quality of life, fatigue, and systemic inflammation (CRP). Inflammation, as observed by imaging, was also improved, with a reduction in MRI scores both at the spine and the SIJ. In addition, a reduction in structural damage at the SIJ was observed with filgotinib. Conversely, results were more limited for the rate of uveitis, or not significant for changes in entheseal score (for filgotinib and upadacitinib compared to placebo). Collectively, these results demonstrated the efficacy of JAKi in key disease manifestations of axSpA, leading to the approval of tofacitinib and upadacitinib in AS by regulatory agencies.

However, additional data are needed to better define the place of these small synthetic drugs in the management of axSpA. First, specific trials in nr-axSpA are required. SELECT-AXIS 2 is a phase-three randomized placebo-controlled double-blind trial evaluating the efficacy and safety of upadacitinib compared to placebo in patients with axSpA, including r-axSpA (study 1) and nr-axSpA (study 2). Study 2 enrolled 314 patients who were randomized to receive upadacitinib for 104 weeks or a placebo for 52 weeks followed by upadacitinib for 52 weeks [44]. The first results of Study 2 showed that upadacitinib met the primary endpoint of ASAS40 at week 14 versus the placebo (45% compared to 23%) [45]. In parallel, studies specifically evaluating patients who do not respond to TNFi and/or IL-17i will be of interest. The effects of these different JAKis on specific skeletal manifestations of SpA, especially enthesitis, require further investigations. In the same way, it remains undetermined whether JAKi may impact acute uveitis or on flares of uveitis in axSpA. In the clinical development of tofacitinib and filgotinib, there was no signal for a higher rate of extra-articular manifestations (uveitis, psoriasis, and inflammatory bowel disease) with JAKi compared to a placebo. To date, clinical trials evaluating the efficacy of JAKi in uveitis have not been conducted. JAKi may improve plaque psoriasis [46], and some of them are licensed for the treatment of ulcerative colitis (tofacitinib). Encouraging results have been observed with upadacitinib in Crohn’s disease [47]. Another relevant question is the impact of such treatment on radiographic progression. Indirect results suggest that filgotinib may improve structural lesions (erosions) at the SIJ, but the effects of JAKi on progressive spinal ossifications remain unknown. Taking into account the control of the IL-23/Th17 pathway by JAK, with downregulation of the production of IL-17 and IL22, it is tempting to speculate that JAKi may probably slow down spinal ossifications. Specific studies with a radiographic outcome (the modified stoke ankylosing spondylitis spinal score (mSASSS)) are therefore required. In order to clarify the role of JAKi in the therapeutic management of axSpA, comparative studies with bDMARDs, especially TNFi, are also required. Finally, since IL-23 signaling involves JAK2 and TYK2 [48], trials with specific/selective inhibitors of JAK2 and/or TYK2 may be relevant for the control of axSpA manifestations. Deucravacitinib is a selective TYK2 inhibitor that has been evaluated in plaque psoriasis [49] and psoriatic arthritis [50] with promising results. However, specific trials in axSpA are not planned. The long-term safety of these new synthetic drugs is another important question in the context of chronic inflammatory disease with treatment over the long term. In clinical trials, the safety profile of the evaluated JAKi was good, with data consistent with results obtained during phase-three trials in RA. Adverse events of interest in patients with axSpA receiving JAKi include infections (especially herpes zoster); venous thromboembolism; and specific laboratory abnormalities, such as elevated CPK or lipid parameters [25]. Concerns have recently emerged regarding the risk of thrombosis under tofacitinib, leading to warnings by the competent authorities. The ORAL Surveillance trial analyzed the safety of tofacitinib (5 and 10 mg twice daily) versus a TNFi in subjects with RA aged 50 years or older who had at least one additional cardiovascular (CV) risk factor. The primary endpoints in this trial were non-inferiority of tofacitinib compared to TNFi in terms of MACE and malignancies. The final analysis of ORAL Surveillance data showed that the non-inferiority criteria were not met for the primary comparison of the combined tofacitinib doses versus TNFi (hazard ratio (HR) for MACE = 1.33 (0.91–1.94); HR for malignancies = 1.48 (1.04–2.09)) [51]. The conclusion is that there was a higher risk of MACE and malignancies with tofacitinib as compared to TNFi in patients with RA who were 50 years of age or older and had at least one additional CV risk factor. Following these results, healthcare professionals were advised to keep considering the benefits and risks of tofacitinib when deciding to prescribe and continue patients on the drug [52]. The Food and Drug Administration (FDA), but not the European Medicines Agency (EMA), extends this recommendation to two other JAKis, namely upadacitinib and baricitinib. The CV risk is increased in axSpA [53], and, thus, caution is certainly required in patients with axSpA and CV comorbidity.

## 7. Conclusions

The JAK/STAT pathway is involved in the signaling of several inflammatory players implicated in the pathogenesis of axSpA. Targeting JAK is thus an attractive and novel therapeutic intervention in this inflammatory rheumatic disease. Clinical trials of JAKi in axSpA have yielded favorable results in key clinical domains of the disease, with an acceptable safety profile. Thus, JAKi could be considered in the therapeutic management of axSpA. Upadacitinib is currently licensed for the treatment of r-axSpA, while EMA has recently approved tofacitinib for axSpA in certain countries. Their place (first, second, or further line of treatment) in the treatment strategy of axSpA remains to be clarified.

## Figures and Tables

**Table 1:** Summary of pivotal trials of Janus kinase inhibitors in axial spondyloarthritis.

Author [Reference]	JAKi	Target	Treatment Arms	Number of Patients	Population	Primary Endpoint	Results
Deodhar [35]	Tofacitinib	JAK1, JAK3 (JAK2)	Placebo Tofacitinib 5 mg × 2/day	269: 136 placebo 133 tofacitinib	AS patients IR ≥2 NSAIDs 80% bDMARD naive 20% IR to ≤2 TNFi	ASAS20 response at week 16	Tofacitinib: 56.4% Placebo: 29.4% *p* < 0.0001
Van der Heijde [38]	Upadacitinib	JAK1	Placebo Upadacitinib 15 mg/day	187: 94 placebo 93 upadacitinib	AS patients IR ≥2 NSAIDs 100% bDMARD naive	ASAS40 response at week 14	Upadacitinib: 52% Placebo: 26% *p* = 0.0003
Van der Heijde [41]	Filgotinib	JAK1	Placebo Filgotinib 200 mg/day	116: 58 filgotinib 58 placebo	AS patients IR ≥2 NSAIDs 93% bDMARD naive upadacitinib group 88% bDMARD naive placebo group	Change from baseline to week 12 in ASDAS score	Filgotinib: −1.47 ± 1.04 Placebo: −0.57 ± 0.82 *p* < 0.0001

IR, inadequate response; AS, ankylosing spondylitis; bDMARDs, biological disease modifying anti-rheumatic drugs; ASAS20/40, assessment of spondyloarthritis society response criteria; ASDAS, ankylosing spondylitis disease activity score.

## Data Availability

Data sharing not applicable.
